# Identification and Quantification of Cell Gas Evolution in Rigid Polyurethane Foams by Novel GCMS Methodology

**DOI:** 10.3390/polym11071192

**Published:** 2019-07-17

**Authors:** Anastasiia Galakhova, Mercedes Santiago-Calvo, Josias Tirado-Mediavilla, Fernando Villafañe, Miguel Ángel Rodríguez-Pérez, Gisbert Riess

**Affiliations:** 1 Chair in Chemistry of Polymeric Materials, Montanuniversität Leoben, Otto Glöckel-Straße 2, A-8700 Leoben, Austria; 2 Cellular Materials Laboratory (CellMat), Condensed Matter Physics Department, Faculty of Science, University of Valladolid, Campus Miguel Delibes, Paseo de Belén 7, 47011 Valladolid, Spain; 3 GIR MIOMeT-IU Cinquima-Química Inorgánica. Faculty of Science, University of Valladolid, Campus Miguel Delibes, Paseo de Belén 7, 47011 Valladolid, Spain

**Keywords:** polyurethane foam, filler, cell gas analysis, gas chromatography-mass spectrometry, thermal conductivity, aging

## Abstract

This paper presents a new methodology based on gas chromatography-mass spectrometry (GCMS) in order to separate and quantify the gases presented inside the cells of rigid polyurethane (RPU) foams. To demonstrate this novel methodology, the gas composition along more than three years of aging is herein determined for two samples: a reference foam and foam with 1.5 wt% of talc. The GCMS method was applied, on one hand, for the accurate determination of C_5_H_10_ and CO_2_ cell gases used as blowing agents and, on the other hand, for N_2_ and O_2_ air gases that diffuse rapidly from the surrounding environment into foam cells. GCMS results showed that CO_2_ leaves foam after 2.5 month (from 21% to 0.03% for reference foam and from 17% to 0.03% for foam with 1.5% talc). C_5_H_10_ deviates during 3.5 months (from 28% up to 39% for reference foam and from 29% up to 36% for foam with talc), then it starts to leave the foam and after 3.5 year its content is 13% for reference and 10% for foam with talc. Air diffuses inside the cells faster for one year (from 51% up to 79% for reference and from 54% up to 81% for foam with talc) and then more slowly for 3.5 years (reaching 86% for reference and 90% for foam with talc). Thus, the fast and simple presented methodology provides valuable information to understand the long-term thermal conductivity of the RPU foams.

## 1. Introduction

Rigid polyurethane (RPU) foams have a closed cell structure with low thermal conductivity, low density, high strength-to-weight ratio, and low moisture permeability. These foams play an important role in many applications as insulation materials of buildings, refrigerators, refrigerated vehicles, piping, and shipbuilding, among other uses [1]. Consequently, the most important property of RPU foams is the thermal conductivity which is determined by three mechanisms [2,3,4]: conduction along the solid phase (λ_s_), conduction through the gas phase (λ_g_) and thermal radiation (λ_r_), with the assumption that convection inside the cells (λ_c_) is negligible because the cell size is small (lower than 2 mm) [5,6]. Since the gas phase of RPU foams represents more than 95% of their volume, λ_g_ supposes a high contribution to the total thermal conductivity (higher than 60%) [7,8]. However, λ_g_ changes with time because insulation gases diffuse out of the foam and atmospheric air diffuses into the foam. Consequently, the total thermal conductivity value increases, this effect being known as foam thermal conductivity aging. Thus, key strategies for improving the thermal properties of foams are the use of blowing agents with low thermal conductivities, or which are able to enhance the overall long-term performance of the foam [9]. For example, C_5_H_10_ remains in the cells for a longer period of time compared to CO_2_, since the latter leaves the foam in the first month after production [10].

The measurement of gas content in PU foams has been rarely considered in the published literature. The reason for that is the complexity of the gas chromatography-mass spectrometry (GCMS) measurement of atmospheric gases. In the 1970s and 1980s there were two categories of cell gas analysis methods: the chemical analysis and those based on physical principles. The chemical analysis was based on dissolving cell gases and performing a titration. The methods based on physical principles included mass spectroscopy, infrared spectroscopy, and gas chromatography [6,7]. In the 1990s and 2000s sampling methods varied, but gas chromatography has generally been used for the gas content measurement. Du Cauze de Nazelle [6] and others [8] studied a gas sample that was taken directly from the foam by a syringe inside desiccator filled with a gas that is not used in the production of the foams and does not interfere with the other gaseous components in the chromatographic analysis. In other studies the foam cylinders are drilled out of foam, twisted and compressed [9,10,11], or grinded [10,12], and a special device collects the escaping gases into an injection system [13,14].

In the present work, a new fast and simple GCMS method for identification and quantification of cell gas evolution in RPU foams is applied. The used method and its comparison with other methods have been described in previous works [13,14]. According to this method a gas sample is taken directly from the foam by a syringe inside a gas desiccator filled with argon. One of the advantages of this method with directly penetrating the foam is that there is no change in temperature, partial pressure, and volume of cell gases during sampling. Therefore, obtained areas under the peak are used for the calculation of concentration of every cell gas, expressed in mole percentage or volume percentage. Afterwards, the molar fraction of every cell gas is used in the Wassiljewa equation [5,15] for the calculation of the theoretical thermal conductivity of the cell gas mixture. Additionally, the specimen is not crushed during the sampling, which is beneficial for the after-test analysis [13,14].

In a previous work [11] describing the thermal conductivity aging of a series of RPU composite foams blown with water and cyclopentane as blowing agents, the different thermal conductivity evolution with time of a reference sample (without the addition of any particle) and samples containing particles (talc, diatomaceous earth, or non-porous silica) was reported. In the early days after foam production, the samples with particles enhanced the thermal conductivity in comparison with reference material, principally due to the significant decrease in cell size promoted by the particles which reduced the radiative contribution. However, after some time, this first improvement was lost in many samples, which could mainly be due to a very quick diffusion of the gases initially occluded inside the cells.

Considering all the above results, it was decided to quantify the changes in gas composition with time for RPU foams using a novel methodology based on gas chromatography-mass spectrometry. For this purpose, two RPU foams belonging to the previous study [16] were chosen: reference material and that containing 1.5 wt% talc, one of which displayed a quicker evolution of the thermal conductivity with time. 

## 2. Materials and Methods

### 2.1. Materials 

The following RPU foams blown with water and cyclopentane were studied in the present work: a pure material (without particles, obtained as a reference material) and a foam containing 1.5 wt% talc with respect to the final mass of the foam. 

The foams were manufactured using a formulation with three components: polyol, isocyanate and cyclopentane. Elastopor H 1501/1 (OH index 651 mg KOH/g, density 1.07 g cm^−3^, viscosity 650 mPa·s) from BASF Poliuretanos Iberia S.A. (Rubi, Spain) was used as a polyol component which includes polyether polyol, catalysts, stabilizers and water. IsoPMDI 92140 (31.5% NCO, density 1.23 g cm^−3^, viscosity 170–250 mPa·s) from BASF Poliuretanos Iberia S.A. was used as the isocyanate component, which is a polymeric diphenylmethane diisocyanate (pMDI). Cyclopentane (99.9% purity) from Sigma Aldrich (Madrid, Spain) was used as the blowing agent. The proportions of the three components were set at 100/160/13 by weight for the polyol, isocyanate and cyclopentane in order to have a free foaming density of 30 kg/m^3^. Talc used is from Imerys (Graz, Austria), which has a mean particle size of 2 microns.

In the case of the foam containing talc, the first step was to premix talc particles into the polyol component at 250 rpm for 5 min in a plastic cup using mechanical stirring. An overhead stirrer (EUROSTAR Power control-visc P1 from IKA, Staufen, Germany with a 50 mm diameter Lenart disc stirrer was used for this purpose. After that, cyclopentane was mixed with the polyol component at 250 rpm for 3 min. Finally, isocyanate was added to polyol blend with cyclopentane and mixed at 1200 rpm for 10 s. The reactive mixture was poured over a wooden mould with dimensions 35 × 35 × 5 cm where it grows. After two days the foam was demoulded and cut into samples with appropriate dimensions in order to characterize their density, cellular structure, and foaming reaction temperature, as well as to measure their thermal conductivity and gas evolution as a function of time. 

More information about the manufacturing and characterization of these materials can be found in a previous article [11]. 

### 2.2. Foam Characterization

Foam density was determined as described by ASTM D1622/D1622M-14 [12]. Three different samples for each material (30 mm in diameter × 25 mm in height) were measured.

After measuring the densities of the samples, open cell content (OC%) was measured by using a Accupyc II 1340 gas pycnometer from Micromeritics, according to ASTM D6226-10 [13]. 

The cellular structure of the foams was analysed by scanning electron microscopy (SEM) with a JEOL JSM-820 microscope. The growth plane of cured foams was examined by SEM after vacuum coating with a gold monolayer. An image analysis technique [14] was used to determine the main characteristics of the cellular structure: mean cell size in 3D(Φ_3D_) and anisotropy ratio (AR). More than 150 cells of different areas of each sample were used for this analysis. 

The foaming reaction temperature reached during the foaming process was measured in a plastic cup 11.5 cm in diameter and 14 cm in height. In order to obtain the temperature measurements, three type K thermocouples were introduced in the centre of the plastic cup at the following heights from the bottom: 2.0 cm, 6.5 cm and 12.5 cm. The data collected by the thermocouples during the foaming process were registered in a computer. Three experiments were carried out for each sample. The foaming temperature obtained by each system was calculated using the average of the maximum temperatures reached by the three thermocouples in the three experiments.

### 2.3. Measurement of Foam Thermal Conductivity

A Rapid K heat flowmeter from Holometrix was used to measure the thermal conductivity under steady heat flow conditions through the test samples of 30 × 30 × 2.5 cm, in accordance with the UNE12667 method [15]. The measurements were performed at 20 °C, where the temperature gradient ranges from 5 °C in the bottom surface plate to 35 °C in the top surface plate. Several measurements were carried out from the fourth day up to around three years after foam production in order to study the evolution of the thermal behaviour with time. The test samples were stored in an opened plastic bag at room temperature (RT) (~23 °C) and atmospheric pressure (~940 hPa) between measurements. 

### 2.4. Measurement of Foam Cell Gas 

GCMS method was applied to determine the C_5_H_10_ and CO_2_ blowing agent gases and their diffusion to the outside and air gases diffusion inside the foam. For this purpose, the foam samples were produced at CellMat laboratory (Valladolid University, Spain) and delivered to the laboratory of Montanuniversität Leoben (Austria) in PE isolation folia. Afterwards, four RPU foam samples (reference and 1.5% talc foams, from two batches - fresh produced and already 3.5 years aged at RT) each of them in 30 × 30 × 2.5 cm^3^ geometry were cut into rectangular specimens (10 × 8 × 2.5 cm) with a saw. Due to the delivery time of the samples, the first GCMS measurement was performed on the 12th day after foam production. Considering that during GCMS sampling the specimens are penetrated with a needle, specimens with open channels have to be discarded after every experiment, therefore, every new test was performed on a new pair of specimens.

The quantitative determination of the gas phase in PU foam includes two steps: sampling and gas chromatographic analysis. The sampling is much more difficult to perform correctly than the gas chromatographic analysis. The main problem was how to get a representative sample of the cell gas without contamination from the surrounding air gases and this problem was solved. 

#### 2.4.1. Sampling

**Figure 1:**
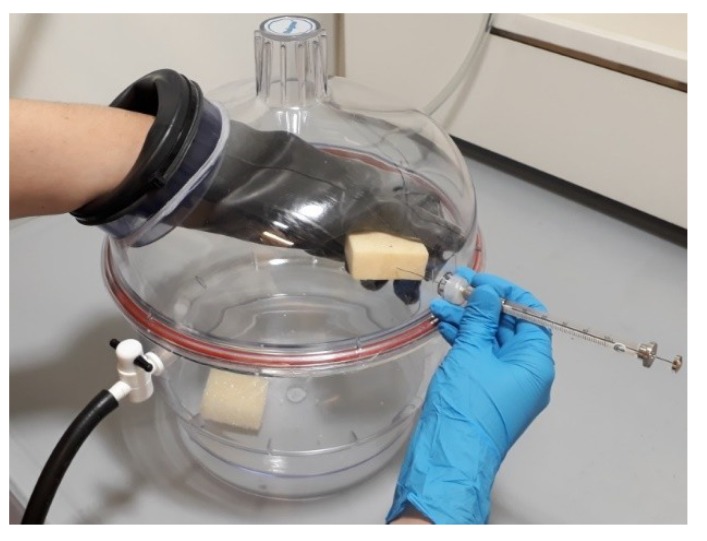
Foam gas sampling.

Seven reference specimens (six fresh produced and one aged during 3.5 years) and seven 1.5% talc specimens (six fresh produced and one aged during 3.5 years) were stored at RT in the PE folia bag for one year of the GCMS testing period. On the day of measurement two specimens (reference and 1.5% talc) were collected from the PE folia bag and placed inside a desiccator, which was flushed afterwards with argon gas in order to remove all air gases surrounding the samples. Argon was chosen because it is not used in foams and does not interfere with the other gaseous components in the chromatographic analysis. The cell gases of the foam were released by a syringe needle that penetrated the foam inside the desiccator (Figure 1). In this sampling care is taken to select the appropriate syringe in order not to contaminate the foam gas sample with air from the surroundings. For this purpose the syringe with push-pull valve with a replaceable needle was selected (the user can select the needle gauge, length and point style in order to achieve the efficient foam penetration with minimized needle coring or blocking). Afterwards, the gas sample was injected into the GC column. The chromatographic analysis was performed.

#### 2.4.2. Chromatographic Analysis

Sample injections were conducted manually by gas tight syringe (SGE, 50R-V-GT). Gas chromatograph-mass spectrometer (GCMS-QP2010 Plus, Shimadzu, Kyoto, Japan) had a fused silica capillary column (length 30 m, ID 0.25 mm). The temperature of column was adjusted to 35 °C. The flow rate of the carrier gas (He) was 104.6 mL/min. SIM mode with five mass fragments (*m*/*z* = 28; 32; 40; 44; 55) was selected.

#### 2.4.3. Determination of Cell Gas Content

The gas content for both samples was accurately calculated with input data obtained from measurement of ratio of each peak area on the chromatograms. In order to know the absolute value of the unknown concentration of gas, there is a need to recalculate the obtained relative value of the unknown concentration of gas according to the calibrated values of the gas of known concentration. For this purpose two calibration mixtures were prepared and measured always on the day of the GCMS foam test [13,14].

#### 2.4.4. Prediction of Thermal Conductivity of the Gas Phase of a PU Foam

Prediction of thermal conductivity of the gas phase in a PU foam is an example of the potential application of the information obtained from the GCMS method. From the cell gas composition, it is possible to calculate the thermal conductivity of the cell gas mixture using the Wassiljewa equation [15]:

(1) $$\lambda_{g}=\;\sum_{i=1}^{n}\frac{y_{i}\lambda_{i}}{\sum_{j=1}^{n}y_{i}A_{ij}},$$

where λ_g_ is the thermal conductivity of the gas mixture, λ_i_ is the thermal conductivity of pure component *i*, (y*_i_*,y*_j_*) are the mole fractions of components *i* and *j* and *A*_ij_ is a function of the binary system that is equal to 1.

In our calculations we have used the following values: At 20 °C, the thermal conductivities of nitrogen (25.40 mW·m^−1^·K^−1^) [16], oxygen (26.11 mW·m^−1^·K^−1^) [16], carbon dioxide (14.50 mW·m^−1^·K^−1^) [10] and cyclopentane (12.00 mW·m^−1^·K^−1^) [17] were used. 

## 3. Results and Discussion

In the previous research [11] the deterioration of thermal conductivity with time was studied for different RPU composite foams, but for understanding the thermal behaviour the analysis of foam gas diffusion was necessary. Therefore, the present study is focused on the determination of gas composition with time. For this purpose, two samples were selected from the earlier study: reference foam and that containing 1.5% talc, one of which showed a faster increase in thermal conductivity at an earlier time.

### 3.1. Morphological Characterization 

The inclusion of talc in the PU formulation had a significant impact on density, cellular structure and foaming reaction temperature (Table 1). The density of the sample containing talc was increased around 4 kg/m^3^, possibly due to the increase in the viscosity of the polyol blend when talc was added. However, open cell content and anisotropy stayed almost unchanged. Instead, the cell size was significantly reduced from around 608 microns for the pure foam to around 307 microns for the material containing 1.5% talc. This cell size reduction of 50% was mainly related to the well-known nucleation effect of the talc which is usually used as a nucleating agent in many types of foams [18]. In addition, the increase in foaming reaction temperature of around 15 °C for foam with talc could favour the cyclopentane evaporation helping to the cell formation and, thus, smaller cells could be formed. The increased foaming reaction temperature for foam with talc could be explained by changes in PU foam reactions due to the interaction between the hydrophobic/hydrophilic surfaces of talc with the PU components, as it has already been observed for other particles incorporated in the PU matrix [19]. 

### 3.2. RPU Foam Cell Gas Composition, Calculated Thermal Conductivity of the Gas Phase and Measured Foam Thermal Conductivity 

#### 3.2.1. Measured Foam Thermal Conductivity

**Figure 2:**
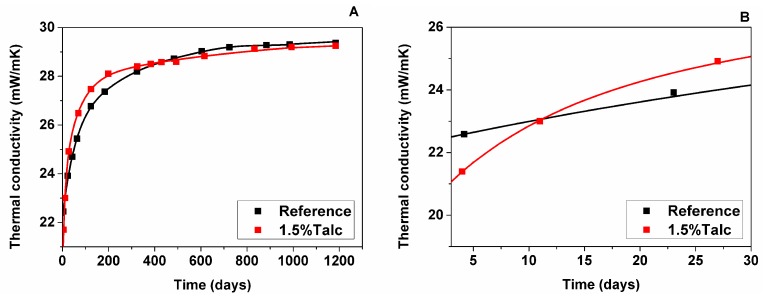
Thermal conductivity evolution for the reference material and for the material with talc: during more than three years (**A**); and during 30 days (**B**).

Thermal conductivity of foam samples was measured over more than three years, as shown in Figure 2A. The samples had a low thermal conductivity at the initial time of the foam production due to their low density (ca. 30 kg/m^3^) and the use of cyclopentane as a physical blowing agent (C_5_H_10_ has a low thermal conductivity and a low diffusion coefficient). However, the thermal behaviour of foam samples get worse with time, reaching around 30 mW/m·K after around 1200 days after foam production because the gases initially occluded inside the cells with low thermal conductivities (14.5 mW/m·K for CO_2_ and 12 mW/m·K for C_5_H_10_ at 20 °C) diffused out, being substituted by atmospheric air with a high thermal conductivity (25.4 mW/m·K for N_2_ and 26.11 mW/m·K for O_2_ at 20 °C). 

In the case of foam with talc, its initial thermal conductivity measured after manufacturing was lower than for the reference foam (Figure 2B). One of the reasons for the obtained lower value was a smaller cell size of foam containing talc (Table 1), which increased the extinction coefficient and, as a consequence, minimized the radiative. However, this improvement in thermal conductivity was not maintained with time for foam with talc since this presented a higher thermal conductivity slope shortly after manufacturing with respect to reference material. However, a lineal relation between the thermal conductivity slope and the foaming temperature was found, so those foams with higher foaming temperature showed a higher thermal conductivity slope, and vice versa [11]. This influence of foaming temperature on the thermal conductivity slope at the initial time is explained in the next paragraph.

The foaming temperature and the gas generated increases the pressure inside cells during the foaming process. Thus, there is a pressure gradient inside and outside the foam cells [5]. The higher foaming temperature reached by the foam with talc could generate a higher pressure gradient inside the cells and, as a consequence, a quicker diffusion of the gasses out of the cells. 

In conclusion, the gas composition measurements with time are fundamental to understanding the differences in the thermal conductivity aging of these two RPU foams.

#### 3.2.2. Foam Cell Gas Composition

**Figure 3:**
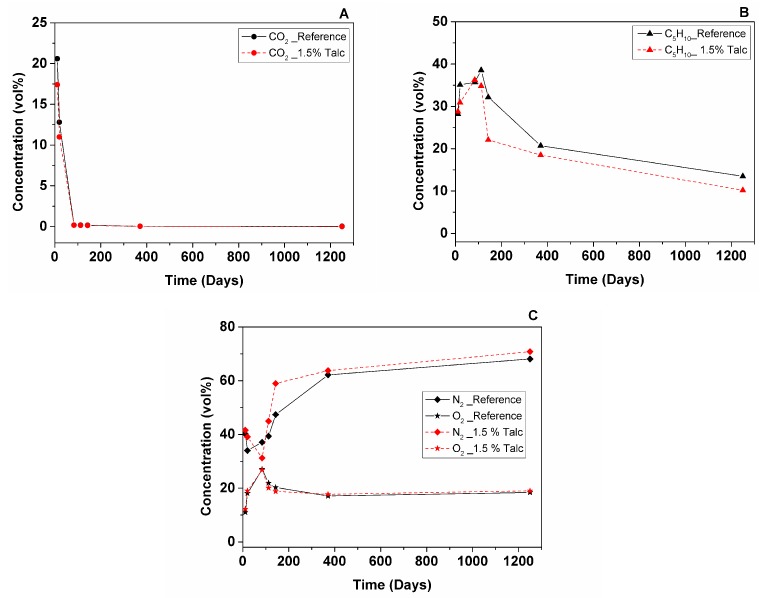
Gas volume percentages with time for the reference material and for the material with talc: CO_2_ (**A**); C_5_H_10_ (**B**); N_2_ and O_2_ (**C**).

**Figure 4:**
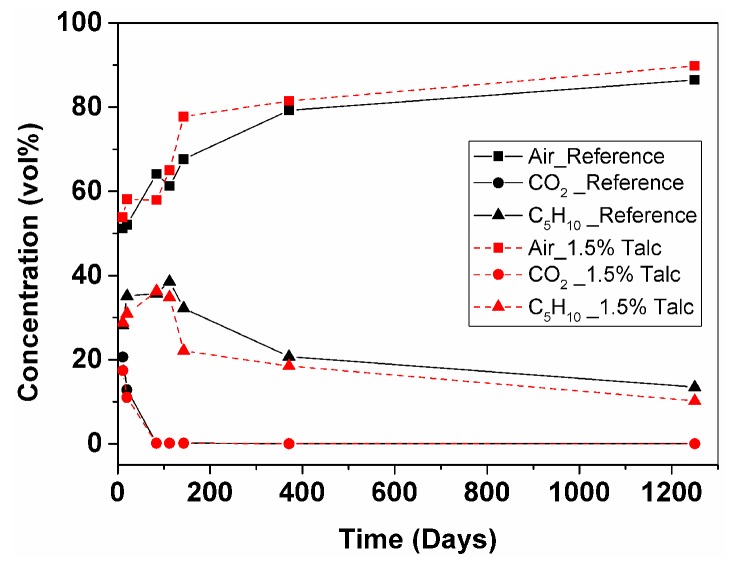
Comparison of gas concentration with time for the reference material and for the material with talc: air (sum of N_2_ and O_2_), CO_2_ and C_5_H_10_.

GCMS test was performed on the 12th and 21st days after manufacturing, and additionally at 2.5, 3.5, 4.5 months, then after 1 and 3.5 years. Unfortunately, testing could not be performed shortly after production due to manufacturing of samples in one country (Valladolid University, Spain) and conducting the test in another country (Montanuniversität Leoben, Austria). The gas composition was accurately calculated with input data obtained from the measurement of the ratio of each peak area on the chromatograms. Table 2 collects the values of the calculated gas volume content and theoretically-derived thermal conductivity of the cell gas mixture by the Wassiljewa equation. Figure 3 and Figure 4 show the evolution of the gas volume percentage with time for both samples.

The initial test of foam thermal conductivity showed a significant difference in results between the reference and that modified with 1.5% talc foams. It would be very representative to show the initial gas composition of both samples after production, taking into account the observed difference in the foaming reaction temperature, which could influence the different evaporation and diffusion of blowing agents into the cells. Unfortunately, it was not possible to perform GCMS test at this stage.

On the 12th day after manufacturing the samples were delivered to the GCMS laboratory and measured. Calculated values of thermal conductivity of the cell gas mixture were similar between the two samples, but for the reference foam it was slightly lower (19.34 mW/m·K) than for the foam with talc (19.70 mW/m·K) due to higher CO_2_ content (20.60 vol% for Reference; 17.40 vol% for Talc) and lower N_2_ and O_2_ content (40.20 vol% (N_2_) and 11 vol% (O_2_)—Reference; 41.60 vol% (N_2_) and 12.20 vol% (O_2_)—Talc). However, C_5_H_10_ content is slightly higher in the foam with talc (28.20 vol%—Reference; 28.80 vol%—Talc) at this stage. The GCMS results are in correspondence with the measured foam thermal conductivity shown in Figure 2B.

Results of gas analysis on the 21st day showed that calculated thermal conductivity of the gas phase of both samples continues to grow (19.48 mW/m·K for Reference; 20.35 mW/m·K for Talc). Both foams showed increased content of C_5_H_10_, what could be explained by liquid cyclopentane that was probably not evaporated completely during foam manufacturing [20] (35.10 vol%—Reference; 30.90 vol%—Talc). CO_2_ is still high, but decreasing gradually (12.80 vol%—Reference; 11.00 vol%—Talc). Air starts to fill the cells, which is shown by the O_2_ content (18.10 vol%—Reference; 19 vol%—Talc), N_2_ content showed a decrease in value, which is not a representative result and is explained by different specimens. Considering that during GCMS sampling the specimens are penetrated with a needle, specimens with open channels have to be discarded after every experiment, therefore, every new test is performed on a new pair of specimens.

After 2.5 months of aging at RT both foams showed an increase in C_5_H_10_ content probably due to their unstabilized condition (35.70 vol%—Reference; 36.25 vol%—Talc). CO_2_ has almost left the foam cells (0.17 vol%—Reference; 0.16 vol%—Talc). Gas phase conduction of foam with talc showed slight improvement (20.03 mW/m·K). Meanwhile, for the reference foam, it has increased (21.39 mW/m·K), showing that the direction of deterioration for both samples became closer to each other, similar to foam thermal conductivity, as shown in Figure 1.

After 3.5 month calculated thermal conductivity of gas phase of both samples showed similar values (20.95 mW/m·K—Reference; 21.46 mW/m·K—Talc), this behaviour looks comparable to trend of measured foam thermal conductivity shown on Figure 1. C_5_H_10_ content continued to grow in the reference sample (38.54 vol%). Meanwhile, it started to leave the 1.5% Talc sample (34.83 vol%). CO_2_ content is relatively stable (0.17 vol%—Reference; 0.14 vol%—Talc).

Results of GCMS at 4.5 months after foam manufacturing showed that stabilization of C_5_H_10_ has finished in both samples and it was leaving the cells (32.19 vol%—Reference; 22.12 vol%—Talc), air was filling the cells (47.37 vol% (N_2_) and 20.28 vol% (O_2_)—Reference; 58.96 vol% (N_2_) and 18.79 vol% (O_2_)—Talc), CO_2_ was decreasing very slowly. Results of gas content measurements showed the deterioration of gas phase conduction for both samples, but for foam with talc it was higher, being the trend similar to the foam thermal conductivity shown in Figure 2A. Thus, the gas diffusion evolved more rapidly in the foam with talc from 3.5 months to 4.5 months after foam manufacturing (Figure 4).

After one year of foam storage thermal conductivity results showed overlapping in values, where the reference foam continued slowly to deteriorate. Although calculated values of the thermal conductivity of the gas phase looked similar, the reference foam showed a slightly lower value (23.26 mW/m·K) than the foam with talc (23.53 mW/m·K). C_5_H_10_ content has gradually decreased but with a different speed: quicker for the reference foam and lower for the foam modified with talc from 4.5 months to one year after foam manufacturing (Figure 3 and Figure 4). Additionally, CO_2_ has almost left the cells (0.038 vol%—Reference; 0.042 vol%—Talc).

After 3.5 years the gas phase contribution to the total foam thermal conductivity showed a similar trend to the one-year results, where the reference foam had a slightly lower value (24.11 mW/m·K) than the foam with talc (24.48 mW/m·K). The lower calculated value of thermal conductivity of the gas phase for the reference foam was explained by a higher content of insulation gases (13.48 vol% C_5_H_10_ and 0.032 vol% CO_2_) and a lower content of air (68.13 vol% N_2_ and 18.35 vol% O_2_) in comparison with talc modified foam. 

Summarizing all of what was said above, the 3.5-year results showed that there is no significant difference between thermal insulation ability of the two samples, but 1.5% talc filler has an influence on the initial value of thermal conductivity. It might be that the longer period of thermal property monitoring is required for evaluation of the difference between two samples, because at 3.5 years the samples have not reached the stationary state yet and, consequently, the thermal conductivity will continue increasing (Figure 2).

## 4. Conclusions

- Considering that the diffusion of gases is one of the mechanisms for deterioration of foam thermal insulation, the measurement of foam thermal conductivity itself does not show the situation with gas composition in every individual foam. Therefore, the GCMS gas content method has shown its benefit as an excellent instrument for the determination of foam cell gas composition and the diffusion of insulation cell gases out of the foam and diffusion of air into the foam cells.
- The presented study was done on two foam types, a reference and one modified with 1.5% talc that have already been described in previous articles, but the gas content method applied in this work made the research of talc incorporation into the foam structure much more comprehensive.
- The sample with talc presented a decrease in the cell size of around 50%, which reduced the radiative contribution to the total thermal conductivity at the initial time of foam manufacturing and, thus, promoted an enhancement of the thermal conductivity. However, that initial thermal improvement shown by foam with 1.5% talc was lost, which has been explained by a higher gas diffusion and, thus, an increase in the thermal conductivity of the cell gas mixture with time.
- GCMS measurement of reference and 1.5% talc modified RPU foams in 10 × 8 × 2.5 cm geometry showed that CO_2_ leaves the foam after 2.5 months (from 21% to 0.03% for the reference foam and from 17% to 0.03% for the foam with 1.5% talc). C_5_H_10_ deviates during 3.5 months, which could be explained by liquid cyclopentane that was probably not evaporated completely during foam manufacturing (from 28% up to 39% for the reference foam and from 29% up to 35% for the foam with 1.5% talc), then it starts to leave the foam and after 3.5 years its content is 13% for the reference and 10% for the foam with talc. Air diffuses inside the cells faster until the one-year point (from 51% up to 79% for the reference and from 54% up to 81% for the foam with talc) and then more slowly until the 3.5-year point (reaching 86% for the reference and 90% for the foam with talc).
- The study of gas mixture content with time could help in the development of the manufacturing of foam with desirable gas compositions and to control the initial thermal conductivity of the foam and, correspondingly, the long-term value.

## Figures and Tables

**Table 1:** Density, open cell content (OC), mean 3D cell size (Φ_3D_), anisotropy (AR) and foaming reaction temperature for the samples under study.

Foam Index	Density (Kg/m^3^)	OC (%)	Φ_3D_ (µm)	AR	Foaming Reaction Temperature (°C)
Reference	31.2 ± 1.7	8.1 ± 1.9	608 ± 68	1.11 ± 0.29	105.9
1.5%Talc	35.6 ± 1.2	9.5 ± 3.1	307 ± 98	1.27 ± 0.27	121.9

**Table 2:** Calculated gas volume content and the theoretically-derived thermal conductivity of the cell gas mixture.

Sample, Index/Storage Time	N_2_, vol%	O_2_, vol%	CO_2_, vol%	C_5_H_10_, vol%	λ_gas calcul_, mW·m^−1^·K^−1^
**Reference**					
12 days	40.20	11.00	20.60	28.20	19.34
21 days	34.00	18.10	12.80	35.10	19.48
85 days (2.5 months)	37.11	27.02	0.17	35.70	21.39
113 days (3.5 months)	39.39	21.89	0.17	38.54	20.95
144 days (4.5 months)	47.37	20.28	0.16	32.19	21.81
372 days (1 year)	62.16	17.08	0.038	20.72	23.26
1250 days (3.5 years)	68.13	18.35	0.032	13.48	24.11
**1.5% Talc**					
12 days	41.60	12.20	17.40	28.80	19.70
21 days	39.10	19.00	11.00	30.90	20.35
85 days (2.5 months)	31.23	26.73	0.16	36.25	21.03
113 days (3.5 months)	44.94	20.09	0.14	34.83	21.46
144 days (4.5 months)	58.96	18.79	0.13	22.12	23.09
372 days (1 year)	63.75	17.73	0.042	18.49	23.53
1250 days (3.5 years)	70.78	19.04	0.030	10.15	24.48
